# Study on the Gelation Process and Mechanical Properties of Organic Polymer Grouting Materials Applied to Fissure Sealing in Underground Mines

**DOI:** 10.3390/polym16040446

**Published:** 2024-02-06

**Authors:** Xuanning Zhang, Ende Wang

**Affiliations:** Department of Geology, College of Resources and Civil Engineering, Northeastern University, Shenyang 110819, China; 1910354@stu.neu.edu.cn

**Keywords:** polyurethane materials, grouting, gelation time, microstructure, density, strength modeling

## Abstract

In this study, organic polymer polyurethane grouting materials were prepared using isocyanate and polyether polyol as the main agents and various additives, the slurry coagulation process was investigated, and the mechanical properties of the polymer samples were tested to explore the influence of the density and soaking time of the polymer on the strength of the samples. The microstructure of the polymer was observed via electron microscopy, and relying on image analysis software, the structural parameters of the polymer cell were analyzed and calculated; the model equation between density and yield strength was established based on the strength model of porous materials developed by Gibson and Ashby. The results show that the initial viscosity and gel time of the polyurethane slurry decrease with the increase of the initial temperature, and the viscosity changes abruptly when the slurry reaches the gel point. The mechanical properties of the polymer increased with increasing density and decreased with increasing soaking time. The interior of the polymer is a porous structure and the pores are approximately spherical; the higher the density of the polymer material, the more uniform the stress distribution of the material, and the higher the percentage of the matrix, which in turn leads to better mechanical properties of the material. The diameter of the polymer cell is negatively correlated with the density, and the model established based on the microscopic parameters of the cell can better predict the yield strength of the polymer. This study helps to deepen the understanding of the microstructure and mechanical properties of polyurethane and provides a certain reference for the application of polyurethane in underground mine reinforcement engineering.

## 1. Introduction

Metallic mineral resources play a very important role in the development of the national economy and the progress of human society, and only by continuously improving the level of development and utilization of metallic mineral resources, can we further accelerate the pace of development of the national economy [1,2]. After a long period of exploitation of surface resources, it is difficult to meet the needs of society, so underground mining efforts continue to increase. As the drilling depth of metal mines continues to increase, the engineering geological environment has become complex and diverse, the rock body internal fissure cross complex, sudden water surges occur frequently, and the safe production of the mine has brought great challenges [3,4]. Grouting technology, as one of the main means of water plugging and reinforcement, has been widely used in water conservancy, transportation, mining, and other engineering fields [5]. Polyurethane grouting material as a kind of green, lightweight organic polymer grouting material is increasingly used in mines and other fields [6].

In underground mine engineering, the gelation process and mechanical properties of the slurry are key factors in determining the effect of grouting. Concerning the problem of fine fissures and groundwater seepage in the rock body of deep underground mines, the traditional cement concrete slurry makes it difficult to penetrate the fine fissures because of its relatively large particle radius; moreover, in the face of the scouring of groundwater flow, the cement concrete slurry can be easily washed away by the water, and it is difficult to form a complete solidified body [7,8,9,10]. Polyurethane slurry is a kind of organic chemical slurry, and compared with traditional inorganic slurry, it has the features of high expansion, strong permeability, good seepage resistance, good durability, short gel curing time, green non-toxic and environmental protection, etc., which can better complete the water plugging and reinforcement work of mining project [11,12]. Polyurethane material is made of isocyanate and polymer polyol as the main reagents, in addition to catalyst, chain extender, surfactant, flame retardant, and other auxiliary reagents mixed [13,14,15].

In recent years, with the gradual increase in the engineering application of polyurethane slurry, the engineering properties of polymer polyurethane slurry have been more widely concerned. Lee JW et al. [16] found that the viscosity characteristics of cement slurry can significantly affect the grouting effect, whereby the higher the viscosity of the slurry, the shorter the penetration distance of the slurry, and the lower the penetration rate. It has been proven that by controlling the viscosity of the slurry, the ideal penetration distance, gel time, and better crack-sealing effect can be obtained. L. Shi and K. Liu et al. [17,18,19,20] investigated the stress–strain behavior, microstructure characteristics, fatigue behavior, and damage mode of polyurethane grouting materials under uniaxial compression, dynamic compression, and cyclic loading. The findings revealed that polyurethane grouting materials with varying densities exhibited either elastic–plastic or atypical brittle properties. Y. He et al. [21,22] investigated how high temperatures and vibrations affected polyurethane foam materials, assessing the degree of damage by analyzing the material’s tensile characteristics after treatment. The findings indicate that as vibration amplitude and duration increase, tensile strength falls and that tiny fractures in the cell structure are the primary cause of vibration damage in polyurethane foams. Stress manifests itself in polyurethane foams when the temperature rises. Stress concentration happens in the polyurethane foam as the temperature rises, and the combined effects of cell wall breakdown and the formation of new fissures cause the tensile strength and modulus of elasticity to drop. G. Gustafson and H. Stille [23] suggested that the relative penetration of the slurry in the fissures is the same, and when the fissure aperture diameter is known, the distance of penetration of the slurry can be estimated. Y. Wang and M. Han [24] conducted a study on the chemical properties of polyurethane polymer materials, compressive and tensile strengths, and explored and analyzed the diffusion pattern of polyurethane polymers in soil and the bonding characteristics with silt. These studies have made great contributions to the understanding of the permeability and mechanical properties of polyurethane, but there are not many studies on the viscosity change law of polyurethane slurry affected by temperature and the factors affecting the mechanical properties of polyurethane slurry.

In this paper, for the fissure water influx and collapse problems encountered in underground mine engineering, a polymer polymerization material suitable for the underground mine environment was formulated, focusing on the viscosity change and mechanical properties of the material. Through the viscosity test of polyurethane, the viscosity of polyurethane was analyzed to change the rule of change with time under different initial temperatures. Mechanical tests were conducted on polyurethane cement to explore the effects of density and soaking time on the strength of the polymer. In order to predict the yield strength of the polyurethane solidification body, the microstructure of the polyurethane cell was observed with a microscope, and the yield strength model of the polyurethane material was deduced based on the microstructural parameters. This study helps to improve the design idea of organic polymer grouting material so that the polyurethane slurry can better serve the underground mine projects.

## 2. Materials and Methods

### 2.1. Grouting Ingredients

The main raw materials that make up the polyurethane grouting material are isocyanate, polyether polyol, or polyester polyol; in addition, there are some auxiliary reagents that also play an important role in the physical properties of polyurethane, and these auxiliary reagents include catalysts, foam stabilizers, chain extender, plasticizers, and so on. The isocyanate and polyether polyol used in this paper are from Siltronic Polyurethane Materials Co. in Zhengzhou, China. The color of isocyanate is black, the viscosity is 0.15 Pa·s, the mass fraction of -NCO is 30%, the color of the polyol is brownish yellow, and the content of -OH is 55 mg KOH/g. The organotin catalyst comes from Runyou Chemical Co., Ltd. in Shenzhen, China. The foam stabilizer dimethyl silicone oil comes from Dalang Tianyu Chemical Materials Company in Dongguan, China. The chain extender and plasticizer are from Yatai United Chemical Co., Ltd. in Wuxi, China. The reagents are shown in Table 1.

### 2.2. Specimen Preparation

**Figure 1:**
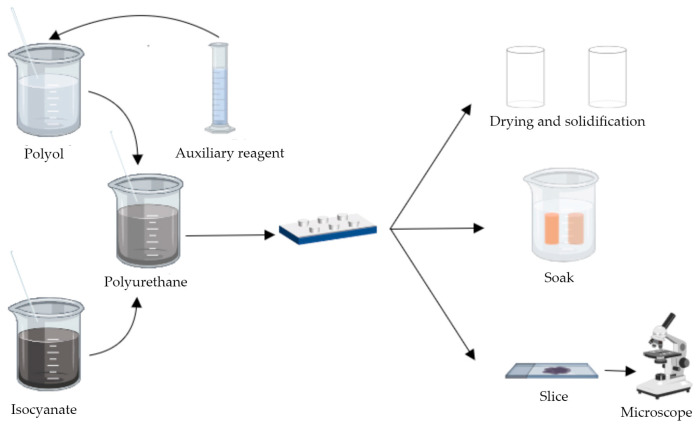
Schematic diagram of polyurethane preparation.

Isocyanate and polyether polyol were mixed according to 1:1 to make a polyurethane prepolymer, and catalyst, foam stabilizer, chain extender, flame retardant, and plasticizer were poured into the prepolymer to obtain a polyurethane slurry. The schematic diagram of the slurry preparation process is shown in Figure 1. The prepared polyurethane slurry was poured into a PVC pipe with a geometric size of 50 × 100 mm and left to stand for 1 h. After the slurry was cured, the sample was taken out of the abrasive and then left to stand for another 24 h, to make a cylindrical solidified body specimen of the polyurethane slurry preparation material.

**Figure 2:**
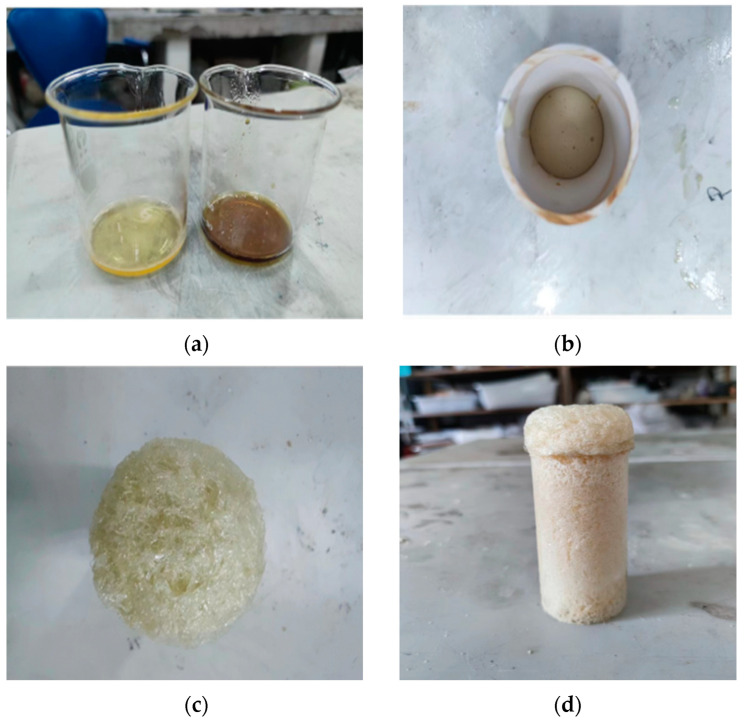
Solidification process of polyurethane slurry. (**a**) Raw material reagents; (**b**) Starting to foam; (**c**) Solidification process; (**d**) Polyurethane consolidation.

By controlling the mass of the poured polyurethane slurry, as well as the volume of the polyurethane solidified body, different densities of the solidified body were obtained, and the solidification process of the polyurethane slurry is shown in Figure 2.

### 2.3. Characterization and Mechanical Tests

The rotational viscometer DNJ-8S produced by Sanuo Instrument Company (Shenzhen, China) is used to measure the viscosity of a polyurethane slurry at different times and a stopwatch is used to record the gel time of the polyurethane slurry. The rotational viscometer is a motor-driven rotor that moves at a constant speed to detect the viscous resistance of the rotor from the liquid to be measured, which is then converted into viscosity. Its usage steps are as follows: First, add an appropriate amount of slurry to the viscometer to ensure that the rotor can be completely immersed in the liquid; Then start the rotational viscometer, at which point the rotor begins to rotate and construct shear stress; Finally, after the reading stabilizes, collect and record the viscosity reading.

The microstructure of the polyurethane solid was observed using a scanning electron microscope, model X-Max-20, from Oxford, UK. Because of the poor electrical conductivity of the polyurethane material, the samples were quenched in liquid nitrogen at low temperatures and sprinkled with gold on the cross-section to improve the electrical conductivity of the surface.

The slurry was poured into a dumbbell-shaped rubber grinding tool coated with a polyurethane release agent, and then the sample was left to solidify before conducting mechanical performance testing. According to the Plastics-Detemination of Compressive Properties, Plastics-Determination of Tensile Properties, and Plastics-Determination of Flexural Properties [25], respectively, the samples were subjected to a compression test, tensile test, and bending test by using a WDW-20 model universal testing machine from Shandong Kece Testing Technology Co., Ltd., Jinan, China.

## 3. Results and Discussion

### 3.1. Viscosity Tests of Polyurethane Slurry

**Figure 3:**
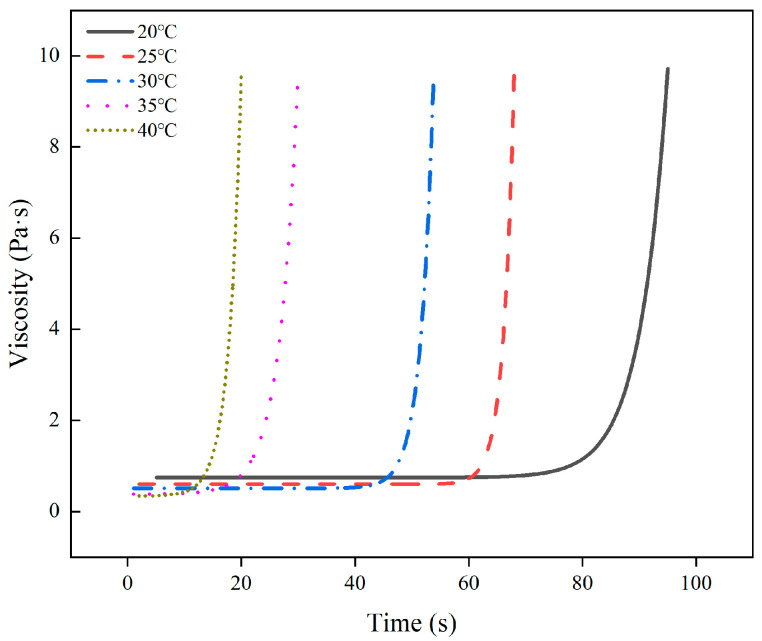
The pattern of change in viscosity with time.

Figure 3 illustrates how the viscosity of the polymer changes over time at various starting temperatures. It is evident that there are two phases to the viscosity change. During the first phase, the polymer’s viscosity remains relatively consistent before gelation, making it a stable property. The gelation point is defined as the inflection point of the curve. In the second stage, following the gelation point, the viscosity increases, and the polymer’s fluidity falls fast.

By fitting the data to the viscosity with time at various temperatures, a model for the viscosity evolution with time was produced. As a function of the fitted curve, the findings demonstrate that the polymer viscosity grows exponentially with time as follows:

(1) $$\eta =K_{1}e^{t/t_{1}}+\eta_{0}$$

where K_1_ and t_1_ are the coefficients, η_0_ is the initial viscosity, t is the time, and η is the viscosity. Table 2 displays the coefficients at various beginning temperatures. All correlation coefficients between the observed data and the fitted equations are more than 0.97799, suggesting that the model performs a superior job of explaining the evolution of polymer viscosity.

**Figure 4:**
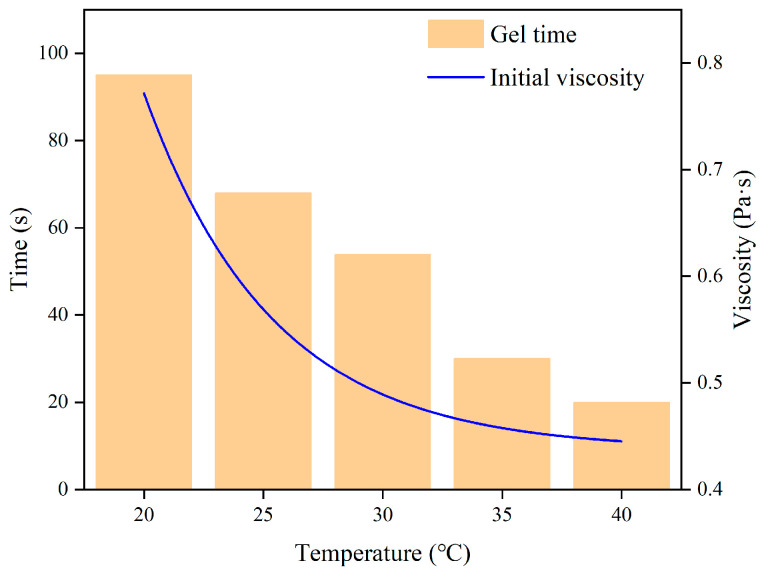
Variation of gelation time and initial viscosity with temperature.

Figure 4 illustrates how the initial viscosity likewise falls exponentially with increasing the initial temperature, and the gel duration rapidly reduces as well. On the one hand, as the temperature rises, the volume of polyurethane expands, the molecular spacing increases and the intermolecular interaction force decreases, which reduces the intermolecular viscous force, and thus the viscosity decreases [26,27,28,29]. On the other hand, the increase in temperature increases the activity of the functional groups in the polyurethane molecules, thus accelerating the curing rate of the polyurethane. Conversely, when the curing temperature is low, the polyurethane molecules are less active, the reaction rate is slower, and the curing time is relatively longer.

### 3.2. Mechanical Properties of Polymers

#### 3.2.1. Effect of Density on Compressive Properties of Polymers

**Figure 5:**
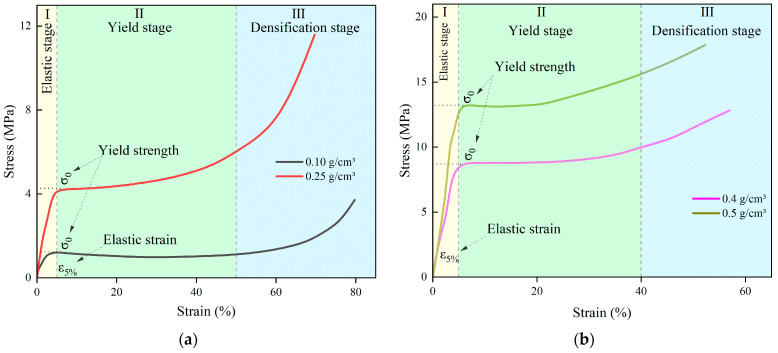
Stress–strain curves for polyurethanes of different densities. (**a**) ρ = 0.1 g/cm^3^, ρ = 0.25 g/cm^3^; (**b**) ρ = 0.4 g/cm^3^, ρ = 0.5 g/cm^3^.

The stress–strain curves of the specimens at different densities are shown in Figure 5. It is evident from the graphic that there are three steps to the process of stress vs. strain. The initial phase is known as the linear elasticity stage, which is represented by the light yellow area in Figure 5. At this stage, the specimen cell is uniformly deformed due to pressure, with the edge of the cell bearing the majority of the load. The deformation of the cell can be recovered after the load is released. The stress is linearly proportionate to the strain, and the strain is less than 5%. This value of 5% strain is typically considered the transition point from elasticity to plasticity of rigid polyurethane grouting material. The material enters the plastic deformation stage, the specimen cell ruptures and collapses, the strain increases, the stress increases slowly or essentially stays the same, and the curve loses its linear characteristics. This is the second stage, known as the yield platform stage, which is represented by the light green area in Figure 5. It can be seen from Figure 5 that in this stage, the strain of the specimen can reach more than 40% under the action of almost constant stress. The third stage is the densification stage, which corresponds to the light blue region in Figure 5, where the cell is almost destroyed as the strain continues to increase, and the stress increases significantly with the strain.

**Figure 6:**
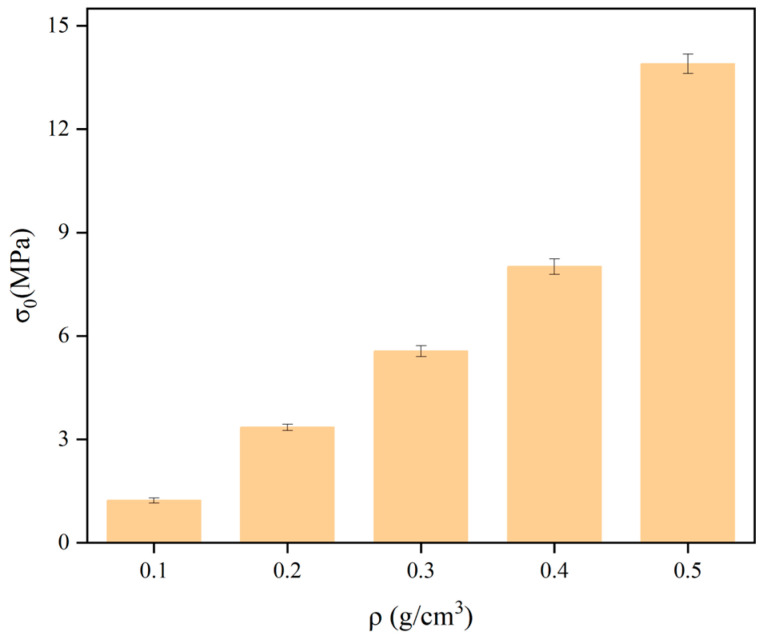
Results of the variation of yield strength with density.

**Figure 7:**
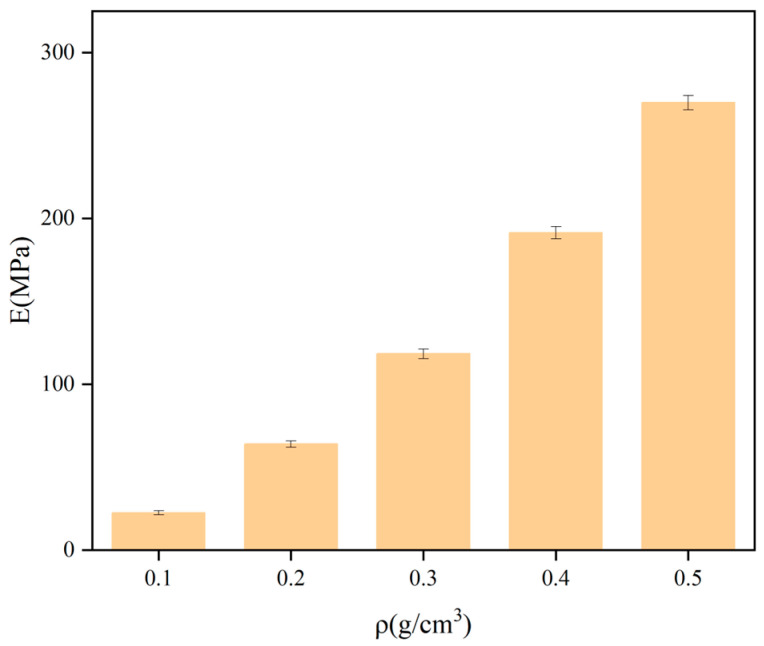
The result of the variation of the modulus of elasticity with density.

**Figure 8:**
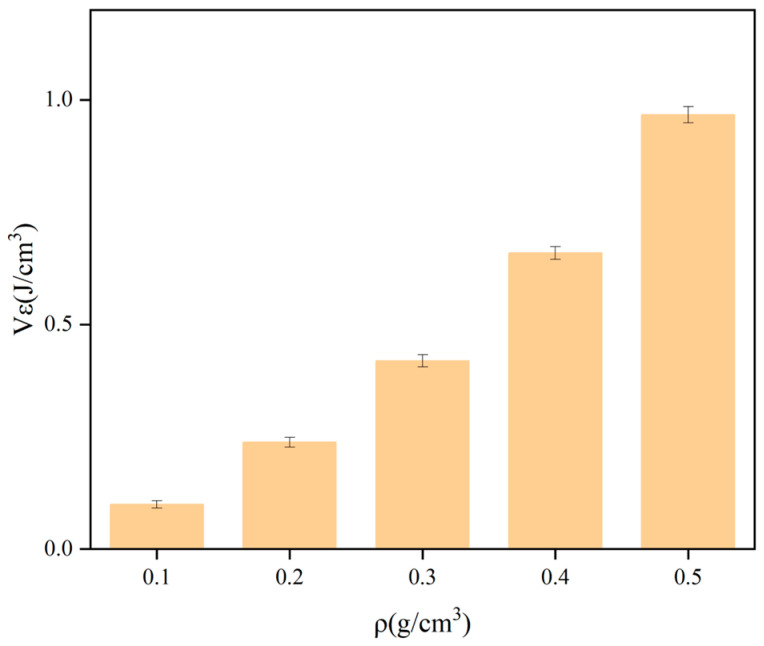
Results of the variation of strain energy with density.

Taking the density ρ as the horizontal coordinate and the yield stress σ, elastic modulus E, and strain energy Vε as the vertical coordinates, the variation rule of the mechanical properties of polyurethane with density is shown in Figure 6, Figure 7 and Figure 8. The stress under 5% strain is regarded as the yield stress of rigid polyurethane, and the rate of change of stress with strain under the elastic phase is taken as the elastic modulus of the sample. After the sample is deformed by the force, the accumulated energy will be generated inside, which is called strain energy, and the calculation formula of strain energy is shown in Equation (2), where ε_0_ indicates the maximum elastic strain of the material, and σ denotes the stress at the time of reaching the maximum elastic strain.

(2) $$V\varepsilon =\int_{0}^{\varepsilon_{0}}\sigma \cdot d\varepsilon$$

It can be seen from Figure 6, Figure 7 and Figure 8 that when the density of the samples is in the range of 0.1–0.5 g/cm^3^, the range of yield stress is in the range of 1.3–14.8 MPa, the range of modulus of elasticity is in the range of 20–280 MPa, and the range of strain energy is in the range of 0.1–1.1 J/cm^3^. The yield stress, elastic modulus, and strain energy all increase significantly with the increase in density.

The microstructural aspects of polyurethane materials were analyzed to investigate the reasons for the change of its mechanical properties with density, and it was found that the yield damage of polyurethane specimens is caused by the rupture damage of microfoam with poor strength properties. On the microscopic scale, the strength of the foam depends on its wall thickness, shape, and size. In low-density polyurethane samples, the foams are in complete contact with each other and loading causes the foams to squeeze each other, resulting in stretching and rupture of the foam walls. However, in high-density polyurethane samples, the number of solid portions between microfoams increases, and the contact interface between neighboring foams decreases. In addition to stretching and rupture of the foam walls, cracks may extend and penetrate the solid matrix, which together leads to yield damage, and the solid matrix is stronger than the foam walls [30,31]. As a result, high-density polyurethane cements have a high compressive strength.

#### 3.2.2. Effect of Soaking Time on Polymer Strength

**Figure 9:**
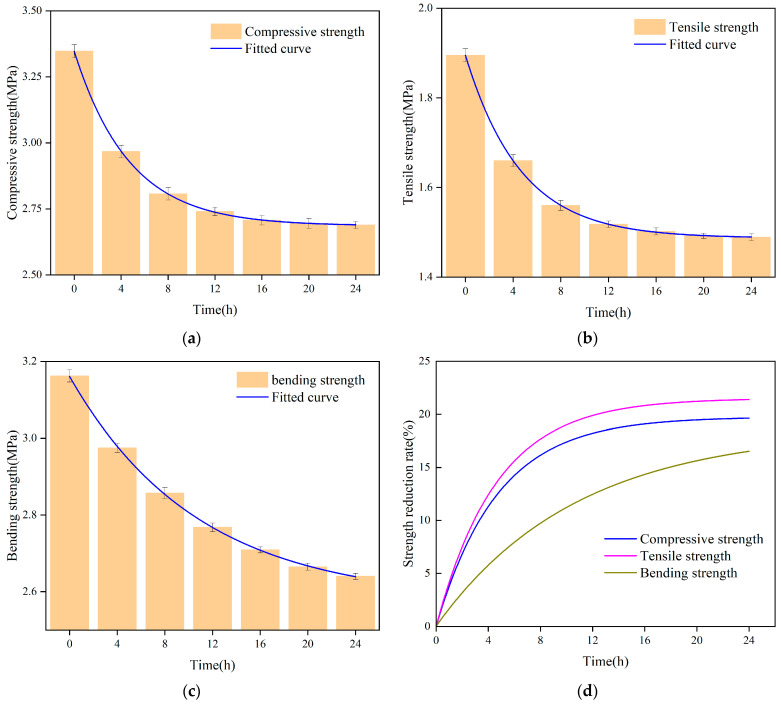
Effect of immersion time on mechanical properties of specimens. (**a**) Effect of soaking time on compressive strength; (**b**) Effect of soaking time on tensile strength; (**c**) Effect of soaking time on flexural strength; (**d**) Relationship between strength reduction rate and soaking time.

The samples with varying soaking durations underwent mechanical testing. The samples’ flexural, tensile, and compressive strengths all declined with longer soaking times, as seen in Figure 9. As seen in Figure 9, the connection between all three strengths and soaking time may be fitted as an exponential function.

The data presented in Figure 9 demonstrate that the tensile strength of the sample experiences the greatest reduction, reaching 22%, within 24 h of immersion. The compressive strength is followed by a reduction rate of approximately 18%, while the flexural strength exhibits the lowest reduction rate at around 15%. The polyurethane material experiences a more significant decrease in strength during the initial 8 h immersion period, followed by a slightly lesser reduction from 8 to 16 h, and a further decrease after 20 h. This phenomenon can potentially be attributed to the porous structure of the polyurethane specimens [32]. During the initial stage of immersion in water, the specimen exhibits a notable water absorption capacity and rate. Simultaneously, the introduction of water disrupts the previously stable structure of the dried specimens, resulting in a substantial reduction in their mechanical strength. As the soaking time increases, the internal pore structure of the specimen gradually approaches saturation, leading to a stabilization of its mechanical properties without discernible decline.

**Figure 10:**
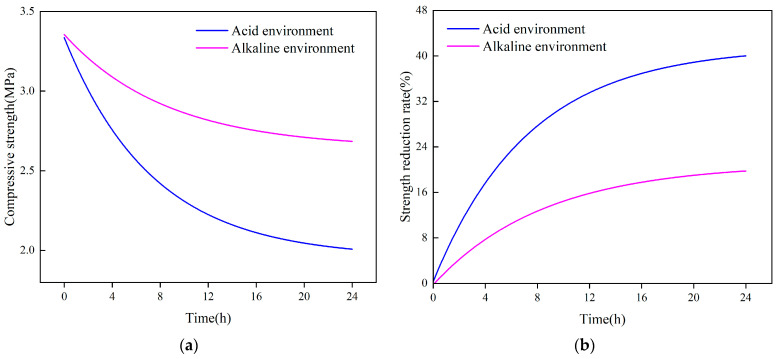
Effect of corrosion time on mechanical properties of specimens. (**a**) Effect of immersion time on compressive strength in different corrosive environments; (**b**) The relationship between the rate of strength reduction and soaking time in a corrosive environment.

The strength changes of the polyurethane samples in an acidic environment at pH 6.5 and in an alkaline environment at pH 8 are shown in Figure 10. The compressive strength of the samples decreased significantly with the increase in soaking time, as can be seen in Figure 10a, where the strength of the samples in an alkaline environment decreases from about 3.4 MPa to about 2.8 MPa after soaking for 24 h, and the strength of the samples in the acidic environment decreases from about 3.4 MPa to about 2.2 MPa. It can be found from Figure 10b, that the negative effect of an acidic environment on the strength of the samples is greater than that of an alkaline environment, and the decrease in the strength of the samples in an acidic environment after 24 h is greater than that of an alkaline environment. It can be seen from Figure 10b that the negative effect of an acidic environment on the strength of the samples is greater than that of an alkaline environment, and the decrease of the strength of the samples in an acidic environment is about 40% after 24 h, while the decrease of the strength in the alkaline environment is about 20%. It is evident that, for the same immersion duration, an acidic environment can reduce sample strength more than an alkaline environment. As a result, acidic environments are more detrimental to the mechanical qualities of polyurethane grouting materials than are alkaline environments.

### 3.3. Microscopic Characteristics of Polymers

#### 3.3.1. SEM Images of the Polymer

**Figure 11:**
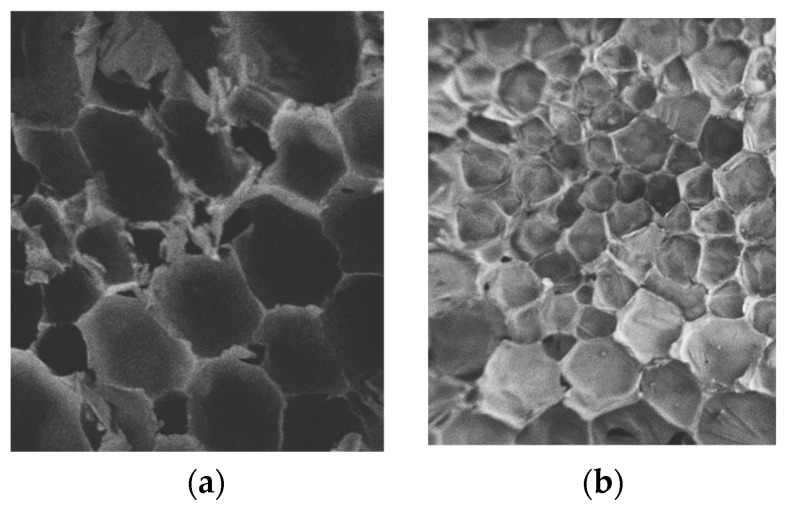
SEM images of the interior of the polymer at different densities. (**a**) ρ = 1.0 g/cm^3^; (**b**) ρ = 3.0 g/cm^3^.

Figure 11 displays the picture captured with a scanning electron microscope of the polyurethane solidified body, illustrating the sample’s appearance at a magnification of 50 times. It is possible to observe that the polyurethane sample is made up of pure solid polyurethane matrix and cell-shaped vesicles on a microscopic level by examining the picture of the specimen’s internal structure. The smaller-density polyurethane specimens have larger cell sizes and relatively irregular cell shapes. The sample’s form grows more circular, the number of cells increases, and the size of the cells reduces as density rises. Unlike the low-density specimens, the stress distribution around the cells in the high-density specimens is more uniform, the stress concentration is reduced, and the matrix accounts for a higher percentage of the sample, which makes the mechanical properties of the high-density samples better than those of the low-density samples.

#### 3.3.2. Characteristic Parameters of Polymer Cell

Polyurethane is a porous material, and the parameters that characterize its cell structure include cell diameter, porosity, etc. The circle in the microscope image represents the cross-section of the cell, but the diameter of the cross-section is not equivalent to the three-dimensional diameter of the cell.

**Figure 12:**
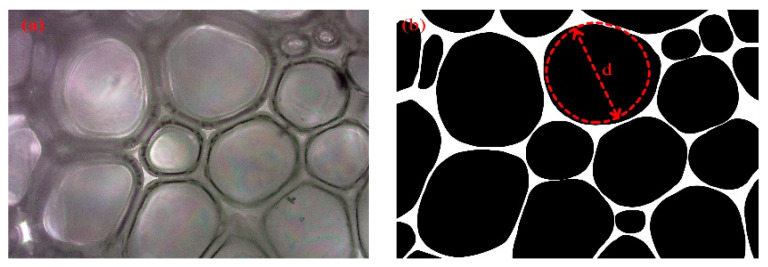
(**a**) Unprocessed microscope image; (**b**) The image after binarization.

The diameter size D of the cell can be expressed by the area A or diameter d of the circular cross-section. To obtain the diameter D of the cell, the diameter d of the circle in the microscope image needs to be counted first, so the microscope image must first be processed:

1. The microscope image was intercepted in the middle part, as shown in Figure 12a;
2. The intercepted image was binarized as shown in Figure 12b;
3. The cross-sectional diameter d of the cell was measured using Image-Pro-Plus software version 6.0.

The cross-section diameter of the cell is the diameter of the circle in the binarized image, as shown in Figure 12b. The average diameter of a circle is shown in Equation (3). Where $\bar{d}$ represents the average diameter of the circle, d_i_ represents the diameter of a single circle, and n_i_ represents the number of circles with a diameter of d_i_.

(3) $$\bar{d}=\frac{\sum n_{i}d_{i}}{\sum n_{i}}$$

**Figure 13:**
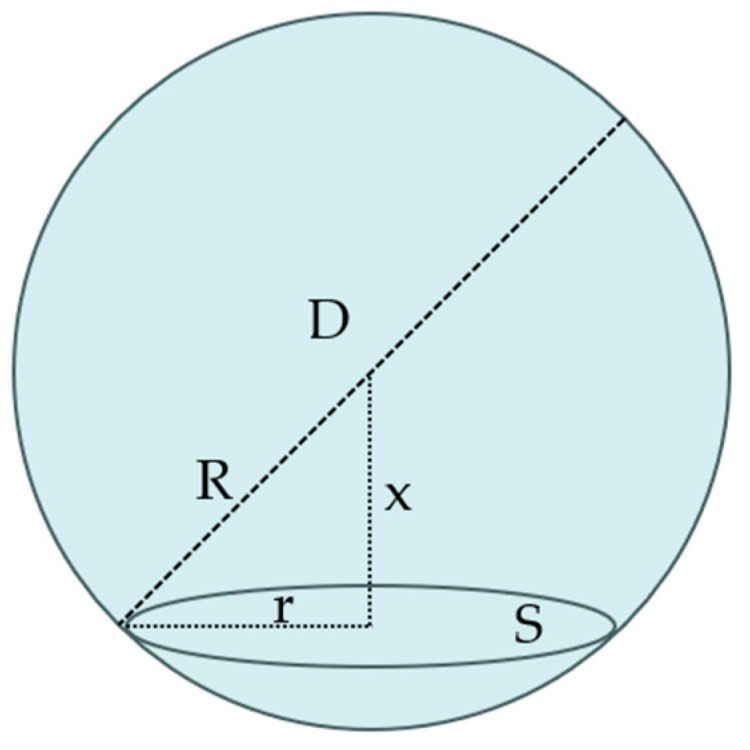
Schematic diagram of structural parameters of polyurethane cell.

Assuming that the cell is a sphere of diameter D, the diameter d obtained from the two-dimensional microscope image is the projected diameter of the cell sphere to the circular cross-section, as shown in Figure 13. The microscope image of the cell is a circular cross-section at a specific distance x from the center of the sphere of the cell. Therefore, the diameter D of the obtained cell can be calculated from the measured diameter d of the circular cross-section, which is calculated as in Equations (4)–(6). Where $\bar{S}$ denotes the average area of the circular cross-section, $\bar{r}$ denotes the average radius of the circular cross-section, and R denotes the radius of the cell.

(4) $$\bar{S}=\pi {\bar{r}}^{2}=\int_{0}^{R}\frac{Sdx}{R}=\frac{1}{R}\int_{0}^{R}\pi \left(R^{2}-x^{2}\right)dx=\frac{2}{3}\pi R^{2}$$

(5) $$D={\left(\frac{6}{\pi }\right)}^{1/2}{\bar{S}}^{1/2}=\frac{\sqrt{6\pi \bar{S}}}{\pi }$$

(6) $$R=\sqrt{\frac{3}{2}}\bar{r}$$

The radii of the circular cross sections were counted using Image-Pro-Plus software, and the radii of the cells with different densities were calculated according to Equation (6), as shown in Table 3.

**Figure 14:**
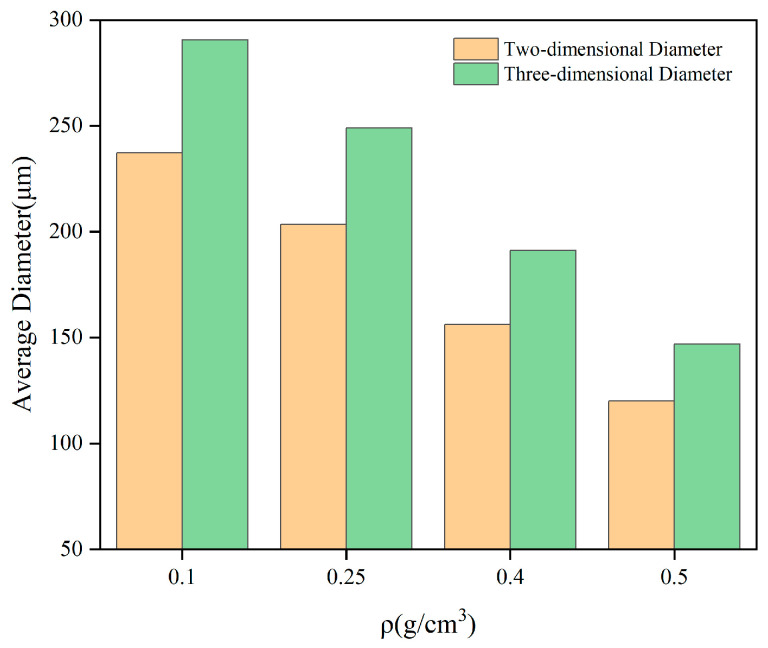
The variation of the diameter of section and cell with density.

**Figure 15:**
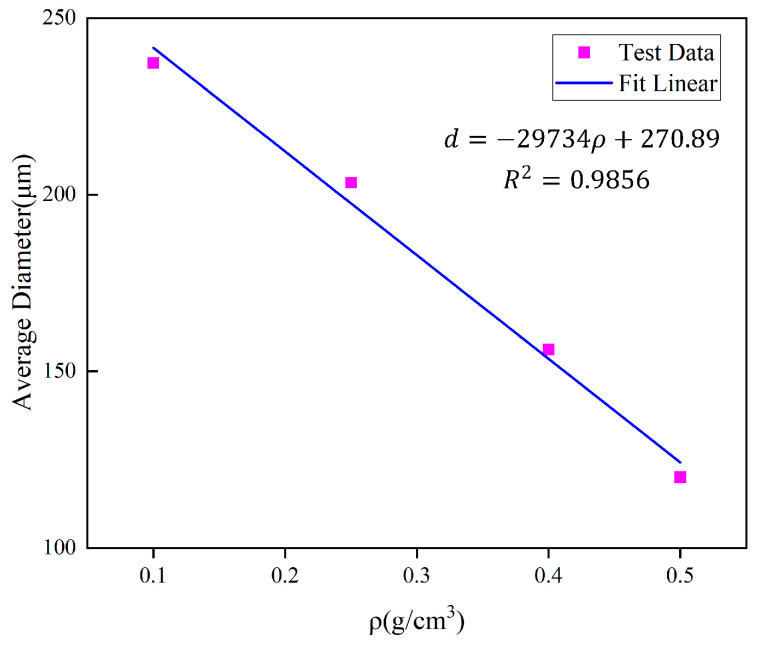
Fitted curve between section diameter and density.

The pattern of change in the diameter of the cross-section circle and the cell diameter with the density is shown in Figure 14, from which it can be seen that the cell diameter decreases as the density increases. A linear fit of the average diameter to the density is shown in Figure 15. The analysis of the relationship between cell diameter and density will help to further understand the effect of polyurethane microstructure on the mechanical properties of the material.

### 3.4. Model of Compressive Strength

#### 3.4.1. Modeling of Strength–Density Relationship

Gibson and Ashby [33] derived the semi-analytical equations for the basic mechanical properties of rigid foams concerning density based on the Gibson and Ashby model, which was developed based on the proposed mechanical model for open-cell and closed-cell foam materials:

(7) $$\frac{\sigma }{\sigma_{\mathrm{ys}}}=A{\left(\phi \frac{\rho }{\rho_{s}}\right)}^{3/2}+B(1-\phi )\left(\frac{\rho }{\rho_{s}}\right)$$

where σ is the material yield strength, σ_ys_ is the solid matrix yield strength, which is a fixed value, A and B are the fitting parameters, and $\rho_{s}$ is the density of the polyurethane matrix, which takes the value of 1.2 g/cm^3^. ϕ is the fraction of solids occupied by the pore ribs.

The above equation can be simplified as a function of the strength concerning the fraction of solids ϕ occupied by the pore prongs and the density ρ of the specimen:

(8) $$\sigma =C{\left(\phi \frac{\rho }{\rho_{s}}\right)}^{3/2}+D(1-\phi )\frac{\rho }{\rho_{s}}$$

The polymers are distributed on the prongs or the walls of the vesicles, and the prong polymer fraction refers to the ratio of polymers contained in the prongs to the total polymers and is denoted by ϕ. Correspondingly, the vesicle wall polymer fraction is 1 − ϕ, which can be expressed as:

(9) $$1-\phi =k\cdot \frac{f\cdot S_{v}\cdot \delta }{1-f}$$

In the formula, k is the scale factor, which takes the value of 0.5, f is the porosity, where f = 1 − ρ/$\rho_{s}$, and S_v_ is the specific surface area of the cell, S_v_ = 3/R = 6/D. According to the fitting equation of Figure 15, d can be expressed in terms of density, and therefore S_v_ can be expressed in terms of density. δ is the wall thickness of the cell, which takes the value of 1 μm.

The average value of the diameters of the cells with different densities is taken as the characteristic bubble pore diameter of the respective densities, and based on Equation (9), the fraction of solids ϕ accounted for by the pore prongs can be found and substituted into the Gibson–Ashby Equation (8), and the relationship between the variation of compressive strength of the urethane material with density can be derived, as shown in Equation (10).

(10) $$\sigma =38.2{\left(\phi \rho \right)}^{3/2}+19.6(1-\phi )\rho$$

#### 3.4.2. Validation of the Model

Table 4 shows the comparison between the predicted and measured compressive strengths of polymers with different densities, and it is found through validation that the strength model of polymers can predict the compressive strengths of materials better according to their densities, and the overall error is kept within 8 percent.

Through Table 4, it is easy to find that with the increase of density, the compressive strength of the material is rapidly enhanced, and the compressive strength of high-density (0.5 g/cm^3^) polyurethane polymer is more than 10 times that of the low-density material (0.1 g/cm^3^). Given that the density of the material is controlled by the grouting volume, different grouting volumes can be deployed according to different strength requirements, which makes it more convenient and flexible to be applied to the sealing and reinforcement of cracks in underground mining projects.

## 4. Conclusions

In this study, we investigated the variation rule of viscosity of polyurethane paste during gelation, and the effects of density and soaking time on the mechanical properties of polymers, and predicted the yield strength of polymers through modeling. The following conclusions were drawn:

- The initial viscosity and gel time of the polyurethane slurry decreased significantly with the increase of the initial temperature, and the change rule of viscosity with time is not linear. The viscosity of the polyurethane slurry is almost unchanged before the gel time point is reached, and when the gel time point is reached, there is a substantial increase in the viscosity of the slurry, and the gel is cured in a short period;
- Under uniaxial loading, the stress–strain curve of polymers is divided into three stages: elastic deformation stage, plateau stage, and densification stage. The yield strength, Young’s modulus, and strain energy of the polymers increase with increasing density. In both water and corrosive environments, the mechanical strength of the specimens decreased with increasing immersion time and stabilized after the immersion time reached 16 h;
- Polyurethanes have a porous structure and the internal cells are approximately spherical. The structural parameters of the polymer cell are statistically determined by graphical processing techniques to establish a link between cell diameter and density.
- Based on the Gibson–Ashby equation, the modeling equations that can predict the yield strength of polymers from their densities are derived in conjunction with the structural parameters of the cell, making the application of polymers more efficient. These studies help to support and guide the application of polyurethane in mine engineering.

## Figures and Tables

**Table 1:** Test materials and dosage.

Raw Material	Type	Proportion (%)
Isocyanate	MDI	47
Polyether Polyol	GE-220	47
Catalyst	DBTDL	1–5
Foam Stabilizer	PMX-200	0.5–2
Chain Extender	BOP	0.5–1
Plasticizer	DOP	0.5–1

**Table 2:** Parameters of viscosity change model.

T/°C	η_0_/Pa s	K_1_	t_1_
20	0.744	2.756 × 10^−8^	−4.847
25	0.598	1.390 × 10^−15^	−1.867
30	0.511	2.949 × 10^−10^	−2.229
35	0.379	9.318 × 10^−4^	−3.258
40	0.341	8.768 × 10^−4^	−2.157

Note: The T denotes the initial temperature.

**Table 3:** Statistical results of cell parameters.

ρ/g/cm^3^	d/μm	D/μm
0.1	237.25	290.57
0.25	203.39	249.10
0.4	156.18	191.28
0.5	120.06	147.04

Note: ρ is the density of the polymer; d is the diameter of the cross-section circle, d = 2r; and D is the diameter of the polymer cell, D = 2R.

**Table 4:** Comparison of predicted and tested values of strength of polymers.

Density/g/cm^3^	Test Strength/MPa	Predicted Strength/MPa	Error/%
0.1	1.226	1.207	1.5
0.2	3.347	3.277	3.6
0.3	5.598	5.963	6.5
0.4	8.517	9.174	7.7
0.5	14.197	15.247	7.4

## Data Availability

The data presented in this study are available in the article.
